# Severe Hyperphosphatemia in a Patient with Mild Acute Kidney Injury

**DOI:** 10.1155/2021/9962624

**Published:** 2021-05-10

**Authors:** Phuong Chi Pham, Raghu Konanur Ventakaram, Jimmy Pham, Harpreet Sidhu, Nada Bader, Phuong Mai Pham, Phuong Thu Pham

**Affiliations:** ^1^Olive View-UCLA Medical Center, Division of Nephrology, Sylmar, CA, USA; ^2^Greater Los Angeles Veterans Administration, Sepulveda Ambulatory Care Center, Los Angeles, CA, USA; ^3^Ronald Reagan UCLA Medical Center, David Geffen School of Medicine, Kidney Transplant Program, Los Angeles, CA, USA

## Abstract

Hyperphosphatemia may arise from various conditions including exogenous ingestion, extracellular shifts due to cell death or alterations in acid-base status, increased bone resorption, hormonal dysregulations leading to reduced renal excretion, reduced kidney function, or faulty measurement techniques. We herein present a case of a young pregnant woman who presented with mild acute kidney injury (AKI), invasive mucormycosis receiving liposomal amphotericin, and hyperphosphatemia out of proportion to the degree of kidney injury. While the patient was given routine phosphate-binding agent by her primary care team for presumed AKI-associated hyperphosphatemia, a full investigation by the renal consulting team for contributing factors other than kidney injury revealed that she actually had pseudohyperphosphatemia associated with the use of liposomal amphotericin. Erroneous treatment of pseudohyperphosphatemia may have been detrimental to this pregnant patient. A literature review for conditions associated with pseudohyperphosphatemia other than the use of liposomal amphotericin will be discussed.

## 1. Introduction

Hyperphosphatemia may arise from various conditions including exogenous ingestion, extracellular shifts due to cell death or alterations in acid-base status, increased bone resorption, hormonal abnormalities leading to reduced renal excretion, and/or reduced kidney function. Hormonal dysregulations or abnormalities that could lead to hyperphosphatemia include hypervitaminosis D, hypoparathyroidism (including inactivating PTH/PTHrP signaling disorders), or fibroblast growth factor-23 (FGF-23) deficiency or nonfunction [1, 2]. Additionally, hyperphosphatemia may result from minor problems associated with the phosphate-measuring technique or presence of phosphate-containing products in blood samples, referred to as pseudohyperphosphatemia [1, 3]. Although pseudohyperphosphatemia is well-described in the literature, it may be under-recognized in routine practice because, unless severe, mild to moderate hyperphosphatemia lacks immediate life-threatening consequences and, accordingly, likely receives a high threshold for clinical concerns and proper evaluation.

We herein present a case of a young pregnant female who presented with mild acute kidney injury (AKI), invasive mucormycosis receiving liposomal amphotericin, and hyperphosphatemia out of proportion to the degree of kidney injury. While the patient was initiated on a phosphate-binding agent by her primary care team for presumed AKI-associated hyperphosphatemia, a full investigation by the renal consulting team for contributing factors other than kidney injury was performed. A thorough evaluation revealed that the patient actually had pseudohyperphosphatemia associated with the use of liposomal amphotericin and promptly averted erroneous phosphate-lowering therapy. A review of the literature for conditions associated with pseudohyperphosphatemia will be discussed.

## 2. Case Report

### 2.1. Clinical History

A 35-year-old 7-week-pregnant woman with type 1 diabetes mellitus was admitted for a fungating mass involving the clivus and cervical vertebrae, presumed to be invasive mucormycosis. On hospital day 1, the patient was initiated on liposomal amphotericin (10 mg/kg) with normal saline support. On day 4, sulfamethoxazole/trimethoprim and ampicillin/sulbactam were added for methicillin-sensitive *Staphylococcal aureus* and *Prevotella buccae* cultured from the fungating mass. On day 10, the patient was noted to have acute kidney injury (AKI) when her creatinine increased from 0.4-0.5 mg/dL to 1.46 mg/dL in association with a progressive increase in serum phosphorus up to 10.7 mg/dL. Nephrology consulting service was consulted for AKI and concurrent multiple electrolyte abnormalities including hypokalemia, hypomagnesemia, and hyperphosphatemia. Laboratory findings are presented in Table 1. Urinalysis was benign with no proteinuria or blood; 3 white blood cells, less than 1 red blood cell, and 2 squamous cells were noted per high power field. Patient's primary team promptly replaced her potassium and magnesium and started sevelamer carbonate 2400 mg t.i.d with meals for hyperphosphatemia. Other medications included aspirin 81 mg q.d, atorvastatin 80 mg q.d, insulin glargine 40 units q.d, metronidazole 500 mg every 8 hours, midodrine 10 mg q.d, and pantoprazole 40 mg q.d.

Physical exam: temperature 36.7°C, heart rate 66 beats per minute, respiratory rate 17 beats per minute, and blood pressure 98/55 mmHg. Head, neck, heart, lungs, and abdomen exams were all within normal limits. Extremities had no edema or rash. Neurological exam revealed no focal abnormalities.

### 2.2. Additional Investigations

Chart review was notable for a prolonged hypotensive episode on day 6 when patient's blood pressure dropped from a baseline of 100/80 mmHg to 75–85/50–60 mmHg over a duration of 4 hours. Additionally, the patient received a computed tomogram with intravenous contrast on the same day.

The underlying etiology of AKI was relatively straight forward and thought to be multifactorial that could be related to the hypotensive episode, tubulointerstitial injury and/or decreased tubular secretion of creatinine unrelated to kidney injury associated with trimethoprim, contrast-induced kidney injury, amphotericin-induced reduced renal blood flow with or without direct tubular toxicity, and possibly early acute tubulointerstitial disease associated with the use of different antibiotics or pantoprazole [4].

Electrolyte abnormalities including acute onset of hypokalemia and hypomagnesemia were attributed to amphotericin-induced tubular cell membrane injury with resulting electrolyte wasting [4]. The mild metabolic acidosis was attributed to the ongoing AKI and possibly some degree of proton back-flow induced by amphotericin [4]. Mild chronic respiratory alkalosis was attributed to the pregnancy state.

Hyperphosphatemia, however, was thought to be significantly elevated and out of proportion to the degree of kidney injury. A systematic investigation for hyperphosphatemia was performed and is summarized in Table 2. Based on patient's clinical history and laboratory findings, both her pregnancy state and chronic respiratory alkalosis could have contributed to hyperphosphatemia, but only to a mild degree [5, 6]. In a nonpregnant adult female, serum phosphorus generally ranges from 2.5 to 4.3 mg/dL but may increase slightly to 3.1 to 4.6 mg/dL during pregnancy [5]. With chronic respiratory alkalosis, renal resistance to parathyroid has been reported to occur which could lead to hyperphosphatemia and hypocalcemia. Nonetheless, the rise in serum phosphorus would only be expected to be within 20% from baseline [6]. Furthermore, patient's serum calcium level was within normal limits after correction for hypoalbuminemia which likely excludes hypoparathyroidism as a contributing factor.

Discussion with our institutional laboratory staff for the possibility of pseudohyperphosphatemia revealed that our institution uses the Beckman-Coulter DxC 800 instrument to measure phosphorus. Unbeknown to all involved clinicians, this instrument is known to measure falsely elevated phosphorus levels in patients receiving liposomal amphotericin B.

While the Beckman-Coulter analyzer revealed a phosphorus level of 10.7 mg/dl for our patient, measurement of phosphorus from the same blood sample on the Roche Cobas instrument from a nearby medical center resulted in a phosphorus level of 4.8 mg/dL.

Diagnosis: pseudohyperphosphatemia due to intravenous administration of high dose liposomal amphotericin.

### 2.3. Clinical Follow-Up

Phosphate-lowering therapy with sevelamer was promptly discontinued. Patient's AKI slowly improved with fluid support and avoidance of further administration of nephrotoxins. Of interest, within 24 hours of amphotericin switch to micafungin from the infectious disease perspective, patient's phosphorus level returned to 4.0 mg/dL as measured by our own Beckman-Coulter analyzer. The patient was discharged within two weeks with resolution of AKI and normal electrolytes.

## 3. Discussion

Measurement of inorganic phosphate relies on the binding of phosphate anions to the acidified ammonium molybdate to form a yellow molybdenum-phosphate complex that absorbs light at 340 nm. Phosphate concentration is subsequently determined based on the absorbance of molybdenum-phosphate complex with ultraviolet spectrophotometry. Chemical analyzers that use very low pH reagent to acidify ammonium molybdate may also hydrolyze organic phosphate from compounds that are present in the serum, which is then measured as part of serum phosphate concentration. Theoretically, administration of any drug that is formulated with phosphorylated liposomal bilayer may also similarly cause pseudohyperphosphatemia. The extent of pseudohyperphosphatemia, however, would depend on the concentration of the culprit drug [7]. Clinicians are advised to consult with their own institutional laboratory staff for the accuracy in the measurement of phosphate by their instrument when phosphate levels are measured among patients receiving drugs formulated with phospholipids.

Of interest, pseudohyperphosphatemia has also been reported in situations involving paraproteinemia (multiple myeloma, Waldenstrom macroglobulinemia, monoclonal gammopathy of undetermined significance), heparin or alteplase-containing blood samples, hemolysis, and hyperlipidemia [7–9]. The precipitation of paraproteins and associated turbidity caused by the acidic reagent used in the phosphate measurement assay has been suggested to interfere with light absorbance by ultraviolet spectrophotometry and erroneous measurement of phosphate [9]. Similarly, turbidity has also been suggested to be the cause of pseudohyperphosphatemia reported with hyperlipidemia [7–9]. Turbidity alone as the cause of pseudohyperphosphatemia associated with paraproteinemia, however, has been challenged [10]. Excess binding of phosphate to certain paraproteins or the actual physicochemical property of the paraprotein or both have also been implicated as a cause of pseudohyperphosphatemia [11–14]. As for heparin and alteplase-contaminated blood samples, phosphate is therein present as a pH buffer agent [15–17]. In the case of hemolysis or any other forms of cell death including tissue infarction and rhabdomyolysis, hyperphosphatemia may be expected as phosphate is a predominant intracellular anion which is released with cell lysis. In essence, hyperphosphatemia in these conditions is “true” hyperphosphatemia. Although hyperbilirubinemia and prolonged blood storage at cold temperature have also been reported to be associated with pseudohyperphosphatemia, these associations have not been confirmed [9, 18, 19]. For the case report on hyperbilirubinemia-associated pseudohyperphosphatemia, concurrent hemolysis was thought to be the underlying etiology rather than the hyperbilirubinemia per se [9].

In summary, any abnormal laboratory finding that is out of proportion to a patient's clinical history deserves a thorough and systematic investigation. The investigation into the hyperphosphatemia out of proportion to the degree of AKI in our current case led to the diagnosis of pseudohyperphosphatemia associated with liposomal amphotericin and averted erroneous phosphate-lowering therapy.  Table 3 summarizes clinical scenarios associated with pseudohyperphosphatemia and the respective reasons for the erroneous laboratory measurement.

## Figures and Tables

**Table 1:** Patient's laboratory findings.

	Creatinine (mg/dL)	Total CO_2_ (mmol/L)	Potassium (meq/L)	Magnesium (mg/dL)	Calcium (mg/dL)/albumin (g/dL)	Phosphorus (mg/dL)
Day 1	0.4	21	3.8	1.9	8.5/3.0	2.6
Day 4	0.5	17	3.1	1.3	8.0/2.0	5.0
Day 6	1.25	17	2.7	1.2	7.8/2.5	6.0
Day 10	1.46	22	3.9 (after repletion)	1.9 (after repletion)	8.1/2.3	10.7

Others: urinalysis was without protein, blood, or cellular casts. Venous blood gas pH 7.36; pCO_2_ 29 mmHg; concurrent chemistry total CO_2_ 17 mmol/L; serum anion gap 7 mmol/L.

**Table 2:** Evaluation of hyperphosphatemia.

Categories	Specific sources	Patient data
*Pseudohyperphosphatemia*	Heparin	Blood drawn by routine phlebotomy not via heparinized central line; patient not on heparin
	Paraproteinemia	Paraproteinemia not checked in a young patient without any suspicious signs/symptoms
	Hyperlipidemia	Total cholesterol 138 mg/dL, low density lipoprotein 71 mg/dl, high density lipoprotein 53 mg/dL, triglycerides 70 mg/dL
	Liposomal amphotericin	Patient was receiving liposomal amphotericin

*Ingestion*	Food source	Patient receives hospital food, hyperphosphatemia from food ingestion is unlikely
	Phosphate-containing medications (e.g., accidental ingestion of phosphate-containing enemas)	

*Gastrointestinal absorption*	Hypervitaminosis D	Vitamin D, 25-OH level 33 pg/mL

*Cellular shift*	Cell death (e.g., rhabdomyolysis, hemolysis, tumor lysis, and bowel infarction)	Clinical exam was benign lactate dehydrogenase 106 U/L and creatinine phosphokinase 21 U/L
	Metabolic acidosis (e.g., lactic acidosis and diabetic ketoacidosis) Chronic respiratory alkalosis	Venous blood gas was consistent with mild normal anion gap metabolic acidosis (which resolved after recovery of kidney function) and chronic respiratory alkalosis. Respiratory alkalosis was likely associated with pregnancy. See discussion in text regarding contribution of patient's acid-base disturbances to hyperphosphatemia.

*Excretion*	Reduced kidney excretion (e.g., GFR <30 ml/min/1.73 m^2^	Patient's estimated GFR >> 30 ml/min/1.73 m^2^
	Hypoparathyroidism, parathyroid hormone resistance	Parathyroid hormone 26 pg/ml; serum calcium was in normal range after correction for hypoalbuminemia. Hypoparathyroidism was unlikely
	Drug-induced (e.g., bisphosphonates) Others: acromegaly, familial tumoral calcinosis, reduced fibroblast growth factor-23 (FGF-23) level or function	Patient was not receiving any bisphosphonates. Conditions such as acromegaly and familial tumoral calcinosis were unlikely contributory due to the acute presentation of hyperphosphatemia. FGF-23 level was also not checked due to low suspicion and diagnosis of pseudohyperphosphatemia was already established.

**Table 3:** Causes of pseudohyperphosphatemia.

Causes	Mechanisms
Liposomal amphotericin (or any drug requiring liposomal bilayer formulation)	Hydrolysis of phosphate from the liposomal bilayer at the low pH used in the assay to measure phosphorus leads to falsely high readings for serum phosphorus
Paraproteinemia (multiple myeloma, Waldenstrom macroglobulinemia, monoclonal gammopathy of undetermined significance)	Precipitation of paraproteins and associated turbidity may interfere with light absorbance by ultraviolet spectrophotometry, thus readings for phosphorus concentration. Excess binding of phosphate to certain paraproteins and specific physiochemical characteristics of paraproteins have also been suggested
Hyperlipidemia	Presumed associated turbidity
Heparin and alteplase-contaminated blood sampling	Heparin and alteplase solutions may contain phosphoric acid as a buffer solution
Hemolysis, rhabdomyolysis, tissue necrosis/infarction	Cellular extrusion of intracellular phosphorus with cell death. This is essentially true hyperphosphatemia

## Data Availability

No data were used to support this case report.
